# Subjective rather than objective patient death experiences link with physicians’ and nurses’ professional quality of life

**DOI:** 10.1186/s12912-024-01724-3

**Published:** 2024-01-15

**Authors:** Chuqian Chen, Jieling Chen

**Affiliations:** 1 Department of Medical Humanities, School of Humanities, Southeast University, Tower B, Humanities Building, Jiulonghu Campus, 211189 Nanjing, China; 2 School of Nursing, Sun Yat-sen University, Guangzhou, China

**Keywords:** Patient death, Professional bereavement, Professional quality of life

## Abstract

**Background:**

Patient deaths are impactful events for professional caregivers in both their professional and personal lives. The present study aims to explore how both subjective and objective patient death experiences are related to various aspects of professional quality of life (ProQOL) among physicians and nurses.

**Methods:**

*S*econdary analyses of cross-sectional data were conducted, and 306 Chinese physicians and nurses whose most recent patient death experience was more than one month prior were included. Objective and subjective patient death experiences were measured based on the number of past patient deaths and the Accumulated Global Changes (AGC) subscale of the Professional Bereavement Scale, respectively. ProQOL was measured with the Professional Quality of Life Scale. Regressions were run following bivariate analyses.

**Results:**

The number of past patient deaths was not significantly linked with any of the three ProQOL scores in either the bivariate analyses or regressions. Meanwhile, higher AGC scores were associated with higher burnout, secondary traumatic stress, and compassion satisfaction scores after participants’ age, occupation (physician/nurse), department, work experience, job commitment, and sense of mission were controlled.

**Conclusion:**

Subjective rather than objective past patient death experiences link significantly with all three aspects of physicians’ and nurses’ ProQOL. The more professional caregivers think that they have been changed by all past patient deaths in their career, the more they experience burnout and secondary traumatic stress, but, the more satisfied they are with their job and the helping itself.

## Background

Patient deaths are impactful events for professional caregivers, and they influence both their professional and personal lives [1, 2]. For physicians and nurses, a patient death can carry both professional meaning, such as suggesting a failure in one’s work, highlighting the limitations of medical science, and a providing case to learn from, and personal meaning, such as indicating the loss of a valuable life and an acquaintance and inspiring empathy for the bereaved family [3]. Shortly after a patient death (approximately one week), bereavement reactions such as grief, frustration, and death anxiety manifest among professional caregivers [4, 5].

In addition to direct short-term reactions, unresolved professional bereavement experiences are related to a series of negative outcomes, such as trauma [6], depression [7], and desire to quit one’s job [8]. According to a study with 828 healthcare workers in Israel during the peak of the COVID-19 outbreak, risks for posttraumatic stress symptoms were higher in COVID-19 wards, where significantly more patient deaths took place, than in non-COVID-19 wards [9]. However, patient death experiences may also contribute to positive outcomes. Among 254 Korean nurses, nursing assistants, social workers, and care workers, the psychological suffering that they experienced after patient deaths was found to be positively linked to posttraumatic growth [10].

Professional quality of life (ProQOL) is “the quality one feels in relation to their work as a helper” [11]. It consists of three aspects, namely, burnout (BO; feelings of unhappiness, disconnectedness, and insensitivity to the work environment), secondary traumatic stress (STS; feelings of being trapped, on edge, exhausted, overwhelmed, and infected by others’ trauma), and compassion satisfaction (CS; feeling satisfied by one’s job and from the helping itself) [11]. Increasing attention has been given to ProQOL among healthcare professionals who frequently experience patient deaths. Among oncology nurses, the prevalence rates of CS, BO, and STS were estimated to be 22.89%, 62.79% and 66.84%, respectively [12]. In emergency department nurses, low to average levels of compassion fatigue and BO and average to high levels of CS were detected [13].

Close links have been found among professional caregivers’ workload, attitudes, perceived competence, and coping methods related to patient deaths and the BO aspect of ProQOL. For instance, delivering death notifications was associated with increased BO rates among American emergency medical service professionals [14]. Among 177 oncologists from Israel and Canada, BO was positively related to the perception of expressing emotion over patient death to be weak and unprofessional [15]. Higher perceived death competence was shown to predict lower BO in Chinese novice oncology nurses [16]. Moreover, grief avoidance among nursing assistants and homecare workers who recently experienced patient death in the U.S. was found to lead to higher risks of BO [14], and psychological education interventions with topics including coping with patient death were found to significantly reduce BO among British doctors [17].

Despite the accumulating evidence that links patient-death-related variables with burnout, studies that straightforwardly checked the associations between professional caregivers’ patient death experiences and ProQOL had failed to detect direct connections. For instance, among 65 pediatric and neonatal intensive care nurses in the U.S., the relationships between experiences of patient death or near death and BO, STS, and CS were all nonsignificant [18]. According to a survey of Israeli palliative care workers, levels of CS, STS, and BO did not differ between the high level of exposure to death group (high-LED; measured by the number of actively dying patients who the participant was exposed to or cared for and the number of hours of direct care for terminally ill patients) and the low-LED group [19].

Previous studies’ failure to detect a direct link may be due to the investigation of objective exposure to rather than subjective experiences of patient deaths as the independent variable. According to constructive views, adverse experiences such as bereavement and trauma are processes of meaning reconstruction, and how people react and cope depends on their perceptions and attributions rather than what happened in reality [20, 21]. Therefore, it is possible that more comprehensive measurements of subjective patient death experiences may help reveal their associations with ProQOL.

Several gaps can be identified in previous explorations of the relationship between patient death experiences and ProQOL. First, attention has been given exclusively to objective exposure to patient deaths as the independent variable, while subjective experiences have been neglected. Second, the research focus has been more on the BO aspect of ProQOL rather than STS and CS. Third, most previous studies were conducted in only one department, which limits the generalizability of the findings. To fill these gaps, the present study used secondary data to answer the following research question: How are subjective and objective patient death experiences related to the BO, STS, and CS component of ProQOL among physicians and nurses from various departments in hospitals?

## Material and methods

### Design

The present study adopted a cross-sectional design. Secondary data analyses were conducted, and the researchers followed the STROBE checklist for reporting cross-sectional studies [22].

In the original project, 563 Chinese urban hospital physicians and nurses who had experienced patient deaths were recruited between August and December 2018, and data were collected through an online survey. Both convenient sampling and snowballing methods were used. Potential participants known personally to the authors of the original study were contacted; in addition, to improve the regional diversity of the sample, advertisements were purposefully sent via a national medical conference whose members included professional caregivers from all over the country. The participants were asked to provide basic information, recall their most recent patient death experience, review their accumulated global changes after all the patient deaths in their career, and rate their ProQOL. Additionally, they were encouraged to communicate the link to friends and colleagues after completing the questionnaire. More details of participant recruitment and data collection are reported elsewhere [23, 24].

### Study participants

The inclusion criteria for the present study were (1) being a physician or nurse whose most recent patient death experience was more than one month before the survey and (2) having a reasonable response time (no shorter than 173 items * 2 s/item = 346 s [25]) for the online survey. In the original project, 72.4% of the participants reported that their most intact memory of the latest patient death faded in less than 1 month [23]. Therefore, the one-month criterion was used to avoid severe distortions of all past patient death experiences and ProQOL by disturbing memories of the most recent patient death. Meanwhile, the response time criterion was used to exclude participants prone to providing careless responses since extremely fast responses indicate a failure to have read, understood, and responded carefully and accurately to survey items [26].

The minimum number of cases needed was calculated with G*Power [27]. For the present study, at least 89 cases were needed for the multiple linear regressions (two tails, *f*^2^ = 0.15, α = 0.05, power = 0.95, maximum number of predictors = 13).

### Instruments

Past patient death experiences, ProQOL, and several control variables were extracted from the original dataset.

For past patient death experiences, the objective measurement was a question asking the number of patient deaths the participant had experienced throughout his or her career (≤ 3, 4–10, 11–20, 21–50, > 50). Meanwhile, the subjective measurement assessed the global changes in the participant’s personal life and professional life as well as ways he or she had faced professional bereavement that was attributed to all patient deaths in his or her career. This construct was measured by the Accumulated Global Changes (AGC) subscale of the Professional Bereavement Scale, a 15-item measurement that was developed and validated in Mandarin. The scale includes factors related to new insights (e.g., “I cherish my life more”), more acceptance of limitations (e.g., “I am more aware of the limitation of medical science”), more death-related anxiety (e.g., “I am more anxious about my own mortality”), less influenced by patient deaths (e.g., “The aftereffects of patient deaths become weaker for me”), and better coping with patient deaths (e.g., “I am better at coping with patient deaths”), which were measured by 4, 3, 4, 2, and 2 items, respectively [23]. Participants were asked to rate “the extent to which you have been changed by all patient deaths in your career in each of the following aspects” on a scale from 0 (no such change or the change was not induced by experiencing patient deaths) to 4 (yes, great deal). Higher AGC scores reflected more intense subjective patient death experiences in general.

ProQOL was measured with the Professional Quality of Life Scale by Stamm [11]. Three subscales were used to assess BO, STS, and CS, each with 10 items [11]. Every item was measured with a 5-point scale. After reverse coding of some items, a higher total score of each subscale showed a higher degree of the construct being measured. With permission from the ProQOL Office, the researchers of the original study slightly modified the expressions in the official simplified Chinese version of the scale.

A series of data on basic demographic information, work-related information, and bereavement-related information were extracted as control variables. Basic demographic information included participants’ age, sex, and religious beliefs. Work-related information consisted of participants’ occupation (nurse/physician), department, hospital level (primary, secondary, tertiary), and work experience (< 1 year, 1–3 years, 4–10 years, 11–20 years, > 20 years). Participants were also asked to rate their commitment to their job and sense of mission in their job on a scale from 0 (extremely weak) to 4 (extremely strong). In terms of bereavement-related information, participants reported their familial bereavement experiences (whether loved ones were lost in their personal life) in the past 2 years and the time since their most recent patient death experience (1–6 months, 6–12 months, > 12 months).

### Analysis process

Analyses were conducted with R packages [28]. Physicians and nurses were grouped together in the analyses, as no significant differences were detected in previous studies between the two groups in either qualitative explorations of their lived experiences of professional bereavement or quantitative measurements of their short-term reactions after each patient death [29]. Following descriptive analyses of all variables, Little’s MCAR test was run to determine whether data were missing at random. Multiple imputation by chained equations [30, 31] was employed to handle missing data, and 5 imputations were run [32]. Afterward, the correlations between BO, STS, and CS were calculated.

Then, bivariate analyses were run to explore the relationships between the three ProQOL scores and all other variables (the 2 variables of patient death experiences and the 11 control variables). Correlational analysis (Pearson’s r), t tests, and ANOVAs were run for continuous, dichotomous, and categorical variables, respectively. Control variables that had significant links with at least one ProQOL score were selected for further regression analyses.

Finally, three multiple linear regressions were run after the assumptions of the linear model (normality, linearity, and heteroscedasticity) were assessed. BO, STS, and CS were the dependent variables. All models used the number of past patient deaths and the ACG score as independent variables, and the same list of control variables was used as well. All dichotomous and categorical predictive variables were turned into dummy variables.

## Results

### Background

A total of 306 participants were involved in the analyses, which met the eligible number for regression. The average age was 32.33 years (*N* = 306, Range: 22–56, SD = 7.23), and the majority of participants were female (*n* = 276, 88.7%) and nurses (*n* = 257, 87.0%). More information is shown in Table 1.

**Table 1:** Background information of participants

Variable	*n* (%)/ *M (SD)*
Age	32.33 (7.23)
Sex	
Male	35 (11.4%)
Female	271 (88.6%)
Occupation	
Physician	49 (16.0%)
Nurse	257 (84.0%)
Department	
Emergency department	16 (5.2%)
Intensive care unit	10 (3.3%)
Oncology	38 (12.4%)
Geriatrics	37 (12.1%)
Surgery	35 (11.4%)
Internal medicine	102 (33.3%)
Operating room	15 (4.9%)
Others (*n* < 10): Palliative care, Pediatrics, Anesthesiology, Obstetrics & Gynecology, Orthopedics, Dialysis, Gastroenterology	53 (17.3%)
Hospital level	
Tertiary	202 (66.0%)
Secondary	93 (30.4)
Primary	11 (2.6%)
Work experience	
< 1 year	9 (2.9%)
1–3 years	40 (13.1%)
4–10 years	147 (48%)
11–20 years	65 (21.1%)
> 20 years	45 (14.7%)
Familial bereavement in the past 2 years	
Yes	166 (54.2%)
No	140 (45.8%)
Having religious beliefs	
Yes	37 (12.1%)
No	269 (87.9%)
Job commitment	3.21 (0.76)
Sense of mission	3.23 (0.81)
Time since the most recent patient death	
1–6 months	159 (52.0%)
6–12 months	52 (17.0%)
> 12 months	95 (31.0%)

Of the 306 participants, 43 and 21 had missing data on objective and subjective past patient death experiences, respectively. Little’s MCAR test showed that the data were missing at random (${\chi }^{2}$ = 45.0, *df* = 44, *p* =.431). No case was deleted due to missing data. The number of past patient deaths were no more than 3, 4–10, 11–20, 21–50, and more than 50 for 62, 88, 41, 51, and 21 participants, respectively. The mean total AGC score was 35.29 (*n* = 285, α = 0.941, *SD* = 14.51). On average, participants scored 17.49 (*n* = 306, α = 0.613, *SD* = 6.19) on the BO subscale, 16.98 on the STS subscale (*n* = 306, α = 0.935, *SD* = 10.43), and 23.22 (*n* = 306, α = 0.937, *SD* = 9.77) on the CS subscale. Positive correlations were found between BO and STS (*r* =.49, *p* < 0.001) and between STS and CS (*r* =.46, *p* < 0.001), while the link between BO and CS was negative (*r* = −.37, *p* < 0.001).

The results of the bivariate analyses between all control variables and the three ProQOL scores are shown in Table 2. Participants’ sex, familial bereavement experiences in the past 2 years, religious beliefs, hospital level, and time since the most patient death did not have significant connections with any of the ProQOL scores. Age, occupation, department, work experience, job commitment, and sense of mission were added along with the number of past patient deaths and the ACG total score into the regressions against ProQOL scores.

**Table 2:** Outcomes of bivariate analyses between all control variables and ProQOL variables

Control variable	Burnout	Secondary traumatic stress	Compassion satisfaction
	Pearson’s *r*		
Age	− 0.13^*^	− 0.03	0.16^**^
Job commitment	− 0.25^***^	0.092	0.39^***^
Sense of mission	− 0.24^***^	0.18^**^	0.44^***^
	*t*		
Sex (female vs. male)	0.78	2.56	0.34
Occupation (nurse vs. physician)	0.99	0.51	-3.39^*^
Familial bereavement in the past 2 years (yes vs. no)	− 0.50	1.60	1.65
Having religious beliefs (yes vs. no)	-1.17	0.82	2.24
	*F*		
Level of hospital	0.30	0.48	0.08
Department	2.58^*^	0.19	1.98
Work experience	2.99^*^	2.89^*^	3.76^**^
Time since the most recent patient death	2.38	0.94	0.08

^*^.01≤ *p* <.05; ^**^.001 ≤ *p* <.01; ^***^p <.001.

For the linear models, the assumptions of linearity (*p* ≥ 0.106) and heteroscedasticity (*p* ≥ 0.476) were met for all three models, while the assumption of normality was only satisfied by the STS model (BO: skewness: *p* =.003, kurtosis: *p* <.001; STS: skewness: *p* =.231, kurtosis: *p* =.970; CS: skewness: *p* =.025, kurtosis: *p* =.311). That is, the data for BO and CS were not normally distributed. According to Schmidt and Finan [33], violations of the normality assumption may not noticeably distort the results as long as the number of observations per variable is larger than 10. Therefore, no transformation was applied to the predicted variable of BO or CS.

### Objective patient death experiences and ProQOL

In the bivariate analyses, the number of past patient deaths was not significantly linked with BO (*F* = 0.42, *p* =.798), STS (*F* = 2.17, *p* =.069), or CS (*F* = 1.11, *p* =.348). Table 3 shows the outcomes of the three multivariate regressions. In the BO (*R*^*2*^ = 0.20, adjusted *R*^*2*^ = 0.14), STS (*R*^*2*^ = 0.39, adjusted *R*^*2*^ = 0.35), and CS (*R*^*2*^ = 0.44, adjusted *R*^*2*^ = 0.40) models, the number of past patient deaths did not significantly link with the predicted variable.

**Table 3:** Outcomes of multivariate regressions (B) against ProQOL variables

	Burnout	Secondary traumatic stress	Compassion satisfaction
Number of past patient deaths (Ref: ≤ 3)			
4–10	-1.03	-1.16	1.65
11–20	0.34	− 0.11	1.77
21–50	-1.60	-2.88	1.14
> 50	− 0.60	-3.71	3.57
AGC	0.09^***^	0.41^***^	0.30^***^
Age	0.02	− 0.05	− 0.01
Occupation (Ref: Physician)			
Nurse	0.13	1.52	− 0.83
Department (Ref: Emergency)			
ICU	-1.52	− 0.62	− 0.17
Oncology	-2.30	− 0.94	2.19
Geriatrics	-4.56^*^	-2.91	3.48
Surgery	-3.80^*^	-1.39	3.33
Internal medicine	-2.63	− 0.82	3.33
Operation room	-4.53^*^	-1.09	2.50
Others (Palliative care, Pediatrics, Anesthesiology, Obstetrics & Gynecology, Orthopedics, Dialysis, Gastroenterology)	-5.02^**^	− 0.31	6.98^**^
Work experience (Ref: > 20 years)			
< 1 year	-1.24	-5.32	-1.61
1–3 years	1.17	− 0.07	1.15
4–10 years	2.10	1.58	0.61
11–20 years	2.45	1.08	− 0.66
Commitment to job	− 0.90	− 0.90	1.35
Sense of mission	-1.49^*^	1.98^*^	3.58^***^

^*^.01≤*p* <.05;^**^.001≤ *p*<.01;; ^***^p <.001.

### Subjective patient death experiences and BO, STS, and CS

In the bivariate analyses, the correlations between AGC and BO (*r* =.16, *p* =.005), AGC and STS (*r* =.58, *p* < 0.001), and AGC and CS (*r* =.49, *p* < 0.001) were all significant. In the multivariate regressions against BO, STS, and CS, higher ACG scores were associated with higher outcome scores. Moreover, a stronger sense of mission was significantly associated with lower BO, higher STS, and higher CS scores. Participants from various departments differed in their BO and CS, and the impacts of age, occupation, work experience, and job commitment on the ProQOL measures were not significant after they were added with the other variables.

## Discussion

### Objective patient death experiences and ProQOL

Using secondary data from 306 Chinese urban hospital physicians and nurses, the present study explored the link between patient death experiences and ProQOL. For the first time, the effects of objective and subjective patient death experiences on ProQOL were separated, and the research question was answered. While objective experiences measured by the number of past patient deaths was not significantly linked with the BO, STS, or CS scores in either the bivariate analyses or regressions, more intense subjective experiences, as reflected by more global changes caused by patient deaths, were associated with higher scores in all three ProQOL aspects.

The failure to detect direct links between healthcare professionals’ objective patient death experiences and ProQOL is consistent with previous findings [18, 19], and the nonsignificant impacts echo a constructive view of people’s experiences: people’s reactions to and coping with adverse events are based not on what actually happened but on their perceptions and interpretations of what happened [20, 21]. For instance, among Hong Kong adults grieving for their loved ones, higher subjective traumatic levels associated with the event were linked with more intense depression and complicated grief, while objective traumatic death (caused by accident or suicide rather than illness or senility) had no relation to either outcome [34].

### Subjective patient death experiences and BO, STS, and CS

Among professional caregivers, the links between subjective stressors and ProQOL have long been established. For both Indian and British nursing staff, perceived stress was found to be positively linked with BO and STS/compassion fatigue and negatively linked with CS [35, 36]. In line with these findings, the present study revealed a clear and direct connection between patient death-specific subjective experiences and ProQOL.

Although BO, STS, and CS reflect both positive and negative aspects of being a professional caregiver, AGC scores are positively associated with all of them. That is, the more that professional caregivers think that they have been changed by all patient deaths in their career, the more they experience BO and STS and the more they are satisfied with their job and with the act of helping others. Similar to the finding that bereavement of loved ones can cause both psychological trauma and posttraumatic growth [37], it seems that profound and in-depth patient death experiences also lead to pain as well as gain.

The link between the AGC score and BO can be explained by the feelings brought about by each patient death. Facing death can be exhausting, especially when deaths are perceived as very impactful [38]. Each patient death can lead to grief, guilt, frustration [39], and, in the Chinese context, worries about potential professional-patient conflicts [2]. Repetitive exposure to such intense feelings may, on the one hand, directly cause profound changes among professional caregivers (as was measured by the AGC) and, on the other hand, indirectly force professional caregivers to become disconnected and insensitive as a strategy for self-protection [40].

Moreover, as reflected in the AGC measurement, many of the changes that are caused by patient deaths are in fundamental dimensions that relate to the meaning of life and death and the value and limitations of medical science. The shattering of basic assumptions is itself a sign of major traumatic experiences [41]. Professional caregivers who report more changes are more likely to be traumatized by patient deaths, and PTSD symptoms correlate positively with vicarious trauma among helping professionals [42].

Meanwhile, when professionals learn more about the uncertainty of life and the limitations of medical science, they cherish the things they can do for their patients out of a “sense of humbleness about their work” [43]. This can result in a higher sense of value attached to the job of helping others. In addition, when professional caregivers gradually learn to be affected less by patient deaths and become better at coping, as was also measured in the AGC, a more internal locus of control can be developed, which is positively linked with CS [44].

Notably, sense of mission was significantly associated with all ProQOL scores: a higher sense of mission was related to less BO, more STS, and more CS. As explained by a Chinese physician in an interview, a sense of mission is a double-edged sword in facing patient deaths: On the one hand, it makes healthcare professionals experience more pain when witnessing patients and their families suffer. On the other hand, it helps them rebound more quickly from such impacts and focus on their duty [2]. The two aspects can explain the links of sense of mission with STS and CS, respectively. Moreover, a professional caregiver with a higher sense of mission may be less likely to become disconnected from patients and insensitive to their pain, despite the challenges that professional bereavement experiences bring. Therefore, their BO risks would be lower.

### Contributions and limitations

This study is the first to separate subjective and objective aspects in the exploration of how patient death experiences are linked to ProQOL. With this approach, a direct and significant link between patient death experiences and ProQOL, which was previously obscured by the exclusive measurement of the number of death events and the neglect of the accumulated changes induced by those events, was revealed for the first time. The findings not only provide a missing piece in the theoretical gap but also further demonstrate how interpretations rather than facts are vital in professional bereavement experiences. Moreover, a comprehensive measurement of ProQOL was adopted, participants from various departments were involved, and a series of control variables were accounted for. All of these factors enhance the generalizability of the findings.

Nonetheless, several limitations exist in the present study. First, a cross-sectional design was adopted, so causal inferences could not be made. Second, convenience sampling was used, and physicians were underrepresented in the present sample, which may have biased the findings. Third, recall bias might have been introduced with the usage of self-reported data.

### Practical implications

The findings of the present study provide practical insights. First, more awareness of patient deaths experiences in healthcare services should be raised for the sake of not only their direct outcomes but also their long-term impacts, such as impaired ProQOL. To prevent patient death experiences from severely interfering with physicians and nurses’ ProQOL, support needs to be provided to those who report being severely influenced by patient deaths rather than those who simply encounter larger numbers of patient death events. Among the targeted population, meaning-centered interventions [45] and education on coping strategies [17] that are adapted to the unique context of patient deaths should be used to deal with threats posed by a sense of disconnection and shattered basic assumptions and to facilitate professionals’ appreciation of the value of the helping profession.

### Future studies

Future studies can use longitudinal designs to reveal clearer causal links between professional caregivers’ patient death experiences and quality of life. More efforts are needed to identify potential mediators and moderators of this relationship to gain more theoretical understanding as well as practical insights. Moreover, a comprehensive framework that involves perceived impacts, perceived competence, values, attitudes, etc., regarding patient deaths can be constructed to form a comprehensive view of all subjective elements of professional bereavement and test how each of the elements contributes to variances in ProQOL when joined with other factors.

## Conclusions

Among Chinese physicians and nurses in the present study, objective and subjective patient death experiences had different links to ProQOL. BO, STS, and CS were all positively related to perceived accumulated global changes caused by patient deaths in regressions with control variables. However, none of the three aspects showed a significant link to the number of past patient deaths.

## Data Availability

The datasets used and/or analysed during the current study are available from the corresponding author on reasonable request.
